# A Novel Case of Biliary Atresia in a Premature Neonate With 1p36 Deletion Syndrome

**DOI:** 10.1177/2324709618790613

**Published:** 2018-07-24

**Authors:** Vonita Chawla, Miran Rhee Anagnost, Alaa-Eldin Eldemerdash, Dahna Reyes, Rebecca Scherr, Kanayo Ezeanolue, Francis Banfro, Rabea Alhosh

**Affiliations:** 1 University of Nevada, Las Vegas, NV, USA; 2 Sunrise Children’s Hospital, Las Vegas, NV, USA; 3 University Medical Center, Las Vegas, NV, USA

**Keywords:** biliary atresia, prematurity, neonate, 1p36 deletion, genetic, Kasai procedure, novel

## Abstract

We describe the case of a premature male neonate diagnosed with biliary atresia who was found to have chromosome 1p36 deletion syndrome. Our patient was born prematurely, at a gestational age of 28 weeks. Pregnancy was complicated by advanced maternal age, gestational hypertension, and intrauterine growth restriction. Physical examination revealed several dysmorphic features, prompting a genetic evaluation, which revealed chromosome 1p36 deletion syndrome. At week 7 of life, he was found to have acholic stools. Direct bilirubin was found to be elevated despite discontinuation of total parenteral nutrition at 3 weeks of life, thus raising the suspicion for biliary atresia. Biliary atresia was confirmed by constellation of clinical, imaging and intraoperative findings. First reported in 1996, 1p36 deletion syndrome has been researched increasingly and several new phenotypic associations have been reported over the years. While attempts at linking specific phenotypic abnormalities with individual gene(s) deletion(s) are being made, deletion patterns that would affect specific organ system or function remain to be fully understood. Thus, clinicians currently rely on reports of previously identified abnormalities. To our knowledge, our patient is the first report of biliary atresia in a patient with chromosome 1p36 deletion syndrome. It is important to determine the etiology of the cholestasis, when present, while caring for premature neonates with 1p36 deletion syndrome. This is necessary to avoid assuming that the cholestasis is arising from total parenteral nutrition administration and not from other gastrointestinal anomalies including biliary atresia, which is a time-sensitive diagnosis.

We describe the case of a premature male neonate diagnosed with chromosome 1p36 deletion syndrome and biliary atresia. Our patient was born prematurely, at a gestational age of 28 weeks to a 41-year-old primigravida mother who had received appropriate prenatal care. Pregnancy was complicated by advanced maternal age, gestational hypertension, and intrauterine growth restriction. Routine prenatal laboratory values were unremarkable. Our patient was delivered electively via a cesarean section, at 28 weeks of gestation, for intrauterine growth restriction. At birth, the patient weighed 660 g, measured 31 cm in length, and had a head circumference of 29 cm. Physical examination revealed hypotonia, frontal bossing, epicanthal folds, depressed nasal bridge, upturned nares, low set posteriorly rotated ears, long philtrum, short neck, bilateral reducible inguinal hernias, bilateral cryptorchidism, crowding of right fourth toe, and left rocker-bottom foot. Our patient had difficulty gaining weight in the neonatal period. Presence of dysmorphic features and failure to thrive prompted a genetic workup. Chromosomal microarray was performed, which revealed a 5.4 MB terminal deletion of the 1p36.33p36.31 locus consistent with chromosome 1p36 deletion syndrome.

At 7 weeks of life, the patient was found to have acholic stools. This prompted a measurement of direct bilirubin, which was found to be elevated at 2.2 mg/dL with total bilirubin measured at 3.5 mg/dL at that time, despite discontinuation of total parenteral nutrition (TPN) at 3 weeks of life. Serial bilirubin measurements showed persistently elevated direct bilirubin (Figure 1), thus raising the suspicion for biliary atresia. The patient underwent an abdominal ultrasound, which failed to visualize a gallbladder. A hepatobiliary iminodiacetic acid scan showed normal hepatic uptake but failed to show uptake in the biliary tree or bowel, within the first hour, at 12 hours, or at 24 hours (Figure 2).

**Figure 1.**
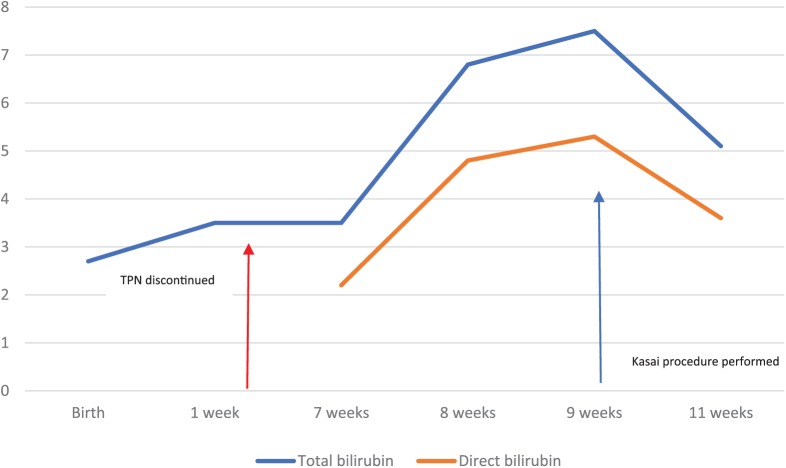
Graphical representation of total and direct bilirubin levels (mg/dL) and age of the patient in weeks.

**Figure 2.**
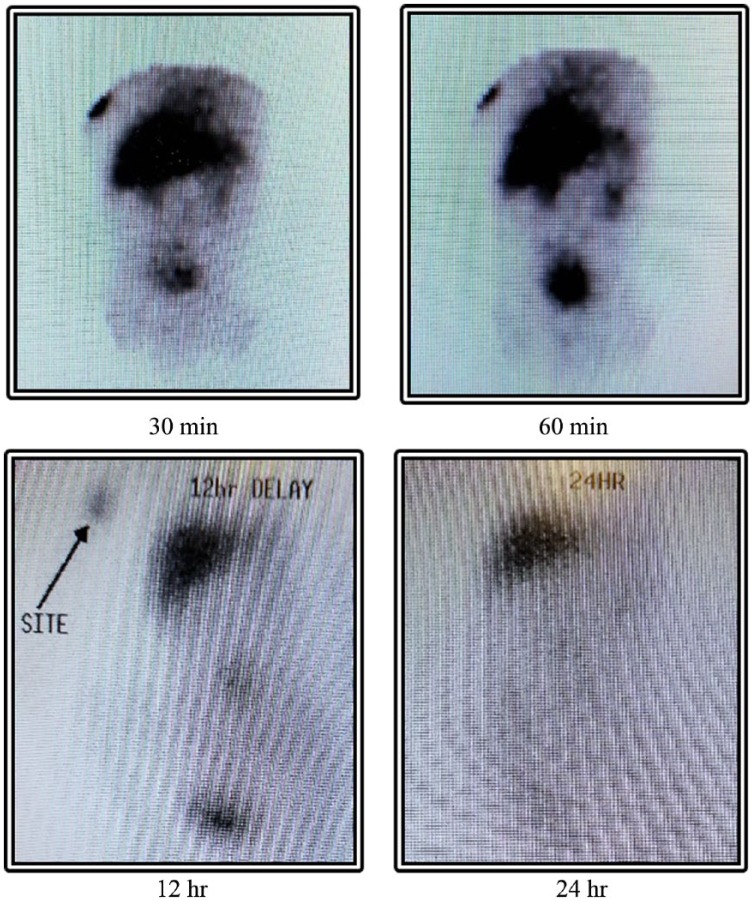
Hepatobiliary iminodiacetic acid scan images at 30 minutes, 60 minutes, 12 hours, and 24 hours showing a lack of biliary tree and/or bowel uptake.

The patient then underwent a liver biopsy, which revealed periportal ductular metaplasia (Figure 3), a finding that may be seen with biliary atresia as well as other nonobstructive causes of cholestasis. Additionally, there was presence of bile pigments in the canaliculi (Figure 4), which can be seen in extrahepatic biliary obstruction such as biliary atresia or from chronic cholestasis arising from TPN administration. While there were no definite histological features to confirm a diagnosis of biliary atresia, the biopsy sample itself was later considered to be low yield, as only a limited number of portal tracts were available for evaluation. After the liver biopsy was obtained, an intraoperative cholangiogram was attempted. However, intraoperatively, only a small diminutive gall bladder was noted. Saline was attempted to be flushed at multiple angles through the fundus of the gall bladder; however, it was impossible to get any flow through the cystic duct. The common bile duct, cystic duct, and common hepatic duct, when examined, appeared to be fibrous cords with lack of lumina with absence of flow proceeding distally. Given the prior remote history of TPN, in addition to the cholangiogram findings, the differential for the features seen on the liver biopsy included TPN cholestasis and/or biliary atresia. The diagnosis of biliary atresia was then made based on constellation of clinical, radiographic, and intraoperative findings, and biopsy findings that can be seen in biliary atresia. Given the confirmatory intraoperative findings of an abnormal biliary tree, a portoenterostomy was performed at this time.

**Figure 3.**
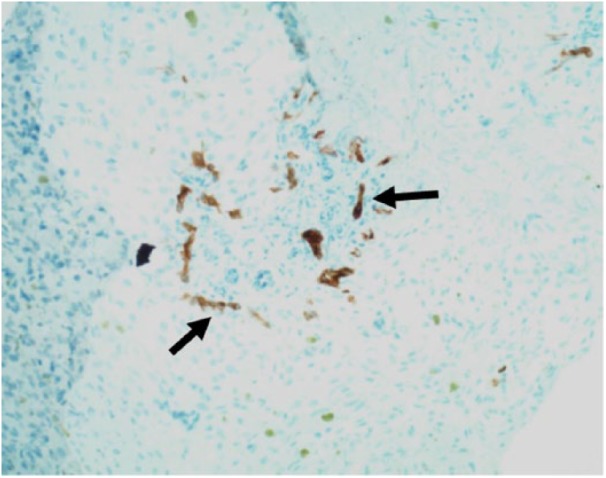
Cytokeratin 7 immunohistochemical stain has been used to highlight areas of ductular metaplasia of the periportal hepatocytes (marked by arrows)—typically seen in chronic cholestatic disease.

**Figure 4.**
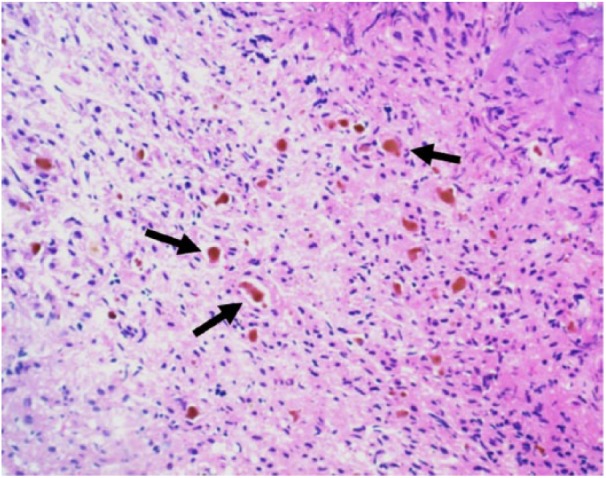
Higher power view of the liver showing canalicular bile pigments (brown pigment within the canaliculi—marked by arrows).

## Discussion

Chromosome 1p36 deletion is a common deletion syndrome with an incidence of 1 in 5000 births.^1^ Since the first report of chromosome 1p36 deletion syndrome in 1996 by Bedell et al,^2^ increasing number of previously unreported phenotypic abnormalities have been identified. While this syndrome has been increasingly studied over the years, linking of specific anatomic and physiologic defects to gene deletions is yet to be fully achieved, leaving clinicians to rely on reports of previously identified abnormalities.^3^

The authors point out recent reports of association of 1p36 deletion syndrome with specific gastrointestinal (GI) abnormalities including intestinal malrotation,^4^ presence of annular pancreas, and anomalous arrangement of pancreaticobiliary duct presenting as pancreatitis, requiring anastomosis between the common hepatic duct and the jejunum,^5^ hepatic steatosis, and bilobed gallbladder.^6,7^ To our knowledge, our patient is the first report of biliary atresia in a patient with chromosome 1p36 deletion syndrome. Currently, there are no reports of genes in the region of chromosome 1p that have been linked to cause biliary atresia, an area that should be explored, given the mounting number of GI abnormalities seen in association with 1p36 deletion syndrome.

Patients with chromosome 1p36 deletion often have failure to thrive, which has historically been attributed to oropharyngeal dysphagia.^8^ However, other anatomical GI abnormalities such as biliary atresia, as seen in our patient, could also contribute to nutritional and weight gain challenges in these patients.

It is also important to determine the etiology of the cholestasis, when present, while caring for premature neonates with 1p36 deletion syndrome. Rashed et al, in 2013, pointed out that ductular proliferation was one of the best indicators of biliary atresia when differentiating biliary atresia from other nonobstructive causes of cholestasis in infants.^9^ In a similar study from 2009, Rastogi et al also found that bile duct proliferation was the strongest indicator of biliary atresia in neonatal cholestasis.^10^ In both these studies, biliary atresia was confirmed by operative findings. As noted by Moreira et al in 2012, liver biopsy may not be conclusive if taken early in the disease process, and repeat interval biopsy may show definitive features of biliary atresia.^11^ This determination is necessary to avoid assuming that the cholestasis is arising from TPN administration and not from other GI anomalies including biliary atresia, which is a time-sensitive diagnosis.
